# 2017 Outstanding Contributions to ISCB Award: Fran Lewitter

**DOI:** 10.1371/journal.pcbi.1005560

**Published:** 2017-06-30

**Authors:** Christiana N. Fogg, Diane E. Kovats, Bonnie Berger

**Affiliations:** 1 Freelance science writer, Kensington, Maryland, United States of America; 2 Department of Mathematics, Massachusetts Institute of Technology (MIT), Cambridge, Massachusetts, United States of America; 3 International Society for Computational Biology, Bethesda, Maryland, United States of America; 4 Computer Science and Artificial Intelligence Laboratory, Massachusetts Institute of Technology (MIT), Cambridge, Massachusetts, United States of America

**Figure:**
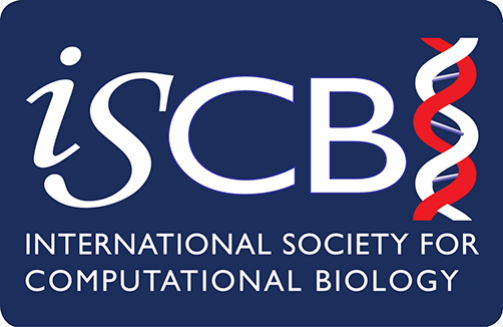


The Outstanding Contributions to ISCB Award was launched in 2015 to recognize individuals who have made lasting and valuable contributions to the society through their leadership, service, and educational work or a combination of these areas. Fran Lewitter is the 2017 winner of the Outstanding Contributions to ISCB Award and will be recognized at the 2017 Intelligent Systems for Molecular Biology (ISMB)/European Conference on Computational Biology meeting in Prague, Czech Republic, which will be held from July 21–25, 2017.

**Fig 1:**
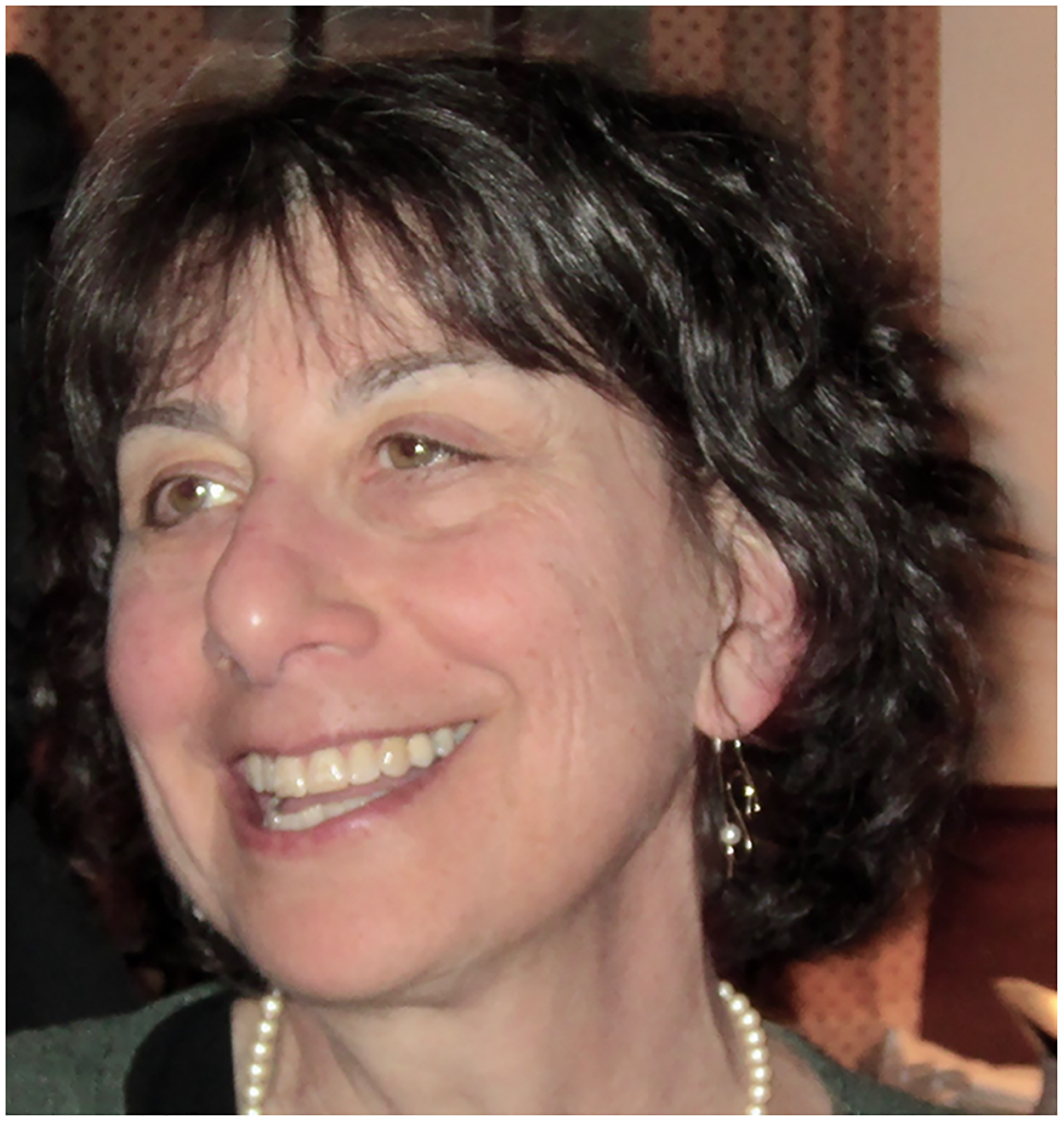
Fran Lewitter.

Fran Lewitter (Fig 1) completed her PhD in human genetics and statistical genetics at the University of Colorado Boulder. After completing postdoctoral work in genetic epidemiology at Harvard Medical School, she worked on the first 5 years of the GenBank project. Lewitter then worked in the biology department at Brandeis University in a number of capacities, including supporting molecular biology computing and being involved with their genetic counseling program. In 1994, she joined the Whitehead Institute for Biomedical Research in Cambridge, Massachusetts, to run a bioinformatics core facility. For 20 years, she worked with and trained basic biomedical researchers who were doing sequencing or were using bioinformatics to gain a deeper understanding of different biological questions. She was later named the Founding Director of Bioinformatics and Research Computing and was given a larger staff as the demand for bioinformatics information grew in the late 1990s and early 2000s.

Lewitter’s first encounter with ISCB occurred when she attended ISMB 2001 in Copenhagen, Denmark, followed by a one-day satellite meeting, Workshop on Education in Bioinformatics (WEB). At the time, Whitehead did not have a large bioinformatics community, and she was in search of peers who were running bioinformatics core facilities and teaching bioinformatics to biologists. “One thing that attracted me to go [to ISMB] was the one-day workshop on education and bioinformatics, since I was so heavily involved in educating people. I went to every meeting since then.” At ISMB 2002 in Edmonton, Lewitter helped organize an informal gathering of bioinformatics core facility managers, and this unique gathering spurred the organization of a mailing list, which became an invaluable resource for Lewitter and her peers as they faced challenges and questions unique to running a core facility.

Since her early encounters with ISCB, Lewitter has become a tireless advocate for bioinformatics education and training on behalf of ISCB. As a core facility director, she has offered her unique academic perspective and voice through her service on the ISCB Education Committee and as a member of the board from 2008–2017. Lewitter recognized the growing demand for bioinformatics training early in her involvement with ISCB, and she worked to strengthen ISCB’s role in supporting bioinformatics education and training by promoting the inclusion of bioinformatics education content in the main conference programs. To this end, she has organized WEBs at ISMB meetings since 2009, and she has helped build ISCB community activities, including the Computational Biology Education Community of Special Interest (CoBE COSI). Lewitter’s leadership of the ISCB Education Committee helped unite the global bioinformatics education community through shared objectives and brought greater awareness of the committee’s work through tutorials and training opportunities offered at ISCB conferences. Lewitter recognizes that one of the most critical aspects of training is “to introduce biologists to bioinformatics vocabulary, whether or not they would be using the primary bioinformatics tools.” This fosters better collaborations between bioinformatics experts and bench scientists and is necessary to facilitate the ongoing integration of bioinformatics into all aspects of biology.

Lewitter has been instrumental in bringing together ISCB and the Global Organization for Bioinformatics Learning, Education and Training (GOBLET) and coordinating activities by which these 2 organizations work together to further bioinformatics training on a global scale. She has advocated for the development and maintenance of bioinformatics education resources on ISCB web pages, and these electronic resources are valuable tools used by the global bioinformatics education community.

Lewitter has valued her membership in ISCB for providing her opportunities to “get to know innovative people.” She has especially appreciated meeting other core facility directors and managers. Lewitter said, “It’s gratifying to hear I am doing the right thing, or other people have ideas that can help me or I can help them. It is good to talk to other people about issues of running a core facility, what courses to teach, or what tools are the best to teach.” Despite having retired from Whitehead Institute 3 years ago, she enjoys her continued involvement in ISCB activities. She is also heartened by the rising generation of ISCB members who are involved with the ISCB Student Council. Lewitter hopes ISCB will continue to grow and thrive and is grateful for being recognized for her steadfast efforts to promote and further bioinformatics education.

